# Polyester Brush Coatings for Circularity: Grafting, Degradation, and Repeated Growth

**DOI:** 10.1021/acs.macromol.3c01601

**Published:** 2023-10-19

**Authors:** Maria Brió Pérez, Mark A. Hempenius, Sissi de Beer, Frederik R. Wurm

**Affiliations:** Sustainable Polymer Chemistry Group, Department of Molecules & Materials, MESA+ Institute for Nanotechnology, Faculty of Science and Technology, University of Twente, P.O. Box 217, 7500 AE Enschede, The Netherlands

## Abstract

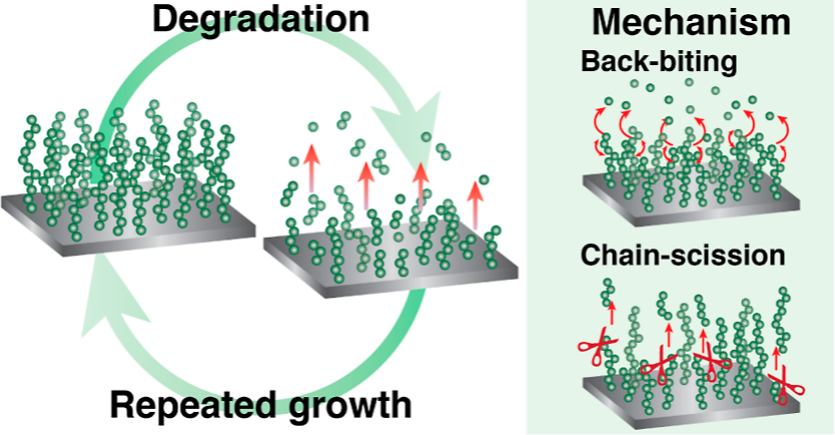

Polymer brushes are widely used as versatile surface modifications. However, most of them are designed to be long-lasting by using nonbiodegradable materials. This generates additional plastic waste and hinders the reusability of substrates. To address this, we present a synthetic strategy for grafting degradable polymer brushes via organocatalytic surface-initiated ring-opening polymerization (SI-ROP) from stable PGMA-based macroinitiators. This yields polyester brush coatings (up to 50 nm in thickness) that hydrolyze with controlled patterns and can be regrown on the same substrate after degradation. We chose polyesters of different hydrolytic stability and degradation mechanism, i.e., poly(lactic acid) (PLA), polycaprolactone (PCL), and polyhydroxybutyrate (PHB), which are grown from poly(glycidyl methacrylate) (PGMA)-based macroinitiators for strong surface binding and initiating site reuse. Brush degradation is monitored via thickness changes in pH-varied buffer solutions and seawater with PHB brushes showing rapid degradation in all solutions. PLA and PCL brushes show higher stability in solutions of up to pH 8, while all coatings fully degrade after 14 days in seawater. These brushes offer surface modifications with well-defined degradation patterns that can be regrown after degradation, making them an interesting alternative to (meth)acrylate-based, nondegradable polymers brushes.

## Introduction

Polymer brushes are dense macromolecular thin films consisting of polymer chains that are attached to a substrate by one of their chain ends.^1^ Polymer brushes can be used to control surface properties, enabling their use as low-friction coatings,^2−6^ drug delivery platforms,^7,8^ or sensors,^9−11^ among others.^12,13^ Generally, brush-based coatings are designed to be long-lasting,^14^ generating additional plastic waste and limiting their applicability in fields such as tissue engineering or drug delivery. To address this, several attempts have been made on the development of degradable polymer brush coatings.^15−18^ For this purpose, aliphatic polyesters are considered as one of the most promising biodegradable polymer types.^19,20^ These include polymers such as poly(lactic acid) (PLA), polycaprolactone (PCL) and polyhydroxybutyrate (PHB), among others. They are currently being used and further studied in bulk as packaging, coatings and in biomedical applications.^21,22^

Although labeled as degradable, these materials do not always fully degrade in real aqueous environments, leading to their undesired accumulation.^23,24^ Thus, it is crucial to understand the degradation mechanism of these polymers under realistic environmental conditions. From a macroscopic perspective, hydrolysis may proceed via either bulk or surface erosion mechanisms, depending on the crystallinity and thickness of the polymer sample. In the case of polyesters that are hydrophobic and semicrystalline, bulk erosion is shown to be predominant. In this case, the rate of diffusion of water exceeds the hydrolysis rate. However, this mechanism may change to surface erosion when hydrolysis occurs more rapidly than water diffusion. This shift will depend on the so-called critical sample thickness (*L*_crit_), above which surface erosion is predominant.^25^

Previous works showed that the hydrolysis of bulk polyesters in aqueous solutions follows distinct molecular pathways under differing degradation conditions. Every polyester type may have a distinct degradation mechanism, which will not only depend on the chemical structure and morphology of the polymer itself but also on external factors such as degradation media, temperature, and pH.^26,27^ In the case of bulk PLA, a backbiting mechanism is predominant in basic conditions, whereas in acidic media, it degrades via chain scission.^28^ Other polymers such as PCL and PHB degrade mainly via random chain scission in both basic and acidic media when synthesized in bulk.^29,30^

These parameters must be taken into consideration when polyesters are used for the development of thin films, such as polymer brush coatings. By having reduced thicknesses, chain confinement, and structural variations, their erosion and degradation mechanisms may vary from the ones observed for the same polymers in bulk. While degradable polyesters are already quite broadly designed and applied in bulk, research on polyester polymer brushes remains scarce. This is partially due to the need of high monomer purity and dry environments during surface-initiated ring opening polymerization (SI-ROP), which makes it a challenging method compared to the typically conducted radical polymerizations.^31^ These have commonly led to PLA and/or PCL polymer brush coatings with ultralow thicknesses (≤10 nm) after long polymerization times.^16,32−34^ A reduced thickness brings difficulties in the characterization of the coating and the degradation process due to a compromised sensitivity on thickness measurements when evaluating changes of a fraction of a nanometer. Furthermore, degrafting reactions must be taken into account. These occur when whole polymer chains are cleaved off via hydrolysis of the surface bonds when these are not sufficiently stable.^35,36^ This can already happen under mild conditions such as humid air.^37^ If the brushes suffer from degrafting, the uncontrolled release of polymer chains would expose the underlying surface, making it susceptible to environmental influences and impeding its reuse for further brush growth due to its irregular structure. Until now, degrafting reactions have been overseen when synthesizing degradable polyester brushes, where weak surface anchors were utilized,^16,32−34^ strongly affecting the stability of the overall coating due to potential cleaving reactions in the anchoring points during degradation essays. Other works show the degradation of brushes in media which is not representative of environmental conditions, where the brush coatings are exposed to extreme environments such as organic solvents or highly alkaline solutions, which also enhance degrafting reactions.^17^ Thus, for degradable polymer brushes, synthetic strategies for stable surface bindings that limit degrafting^14^ are essential. Some of the most relevant surface anchors for stable polymer brush grafting are based on polydopamine (PDA)^38,39^ or poly(glycidyl methacrylate) (PGMA),^40,41^ which strongly bind to various substrates.

In this work, we have developed a synthetic strategy to graft polyester brushes (up to 50 nm) from polyol-based stable macroinitiators on silicon surfaces. Organocatalytic SI-ROP was used to synthesize poly(lactic acid) (PLA), polycaprolactone (PCL) and, for the first time, polyhydroxybutyrate (PHB) polymer brush coatings of varied thicknesses with high control and reproducibility. The polyester brushes were grown from a new synthetic approach of macroinitiators based on TRIS-modified PGMA to prevent undesired brush degrafting. The modified surfaces were confirmed and analyzed by contact angle, Fourier-transform infrared spectroscopy (FTIR) and atomic force microscopy (AFM). After that, the brushes were immersed in solutions of varying pH, and their degradation profile was evaluated with spectroscopic ellipsometry (SE).

The three polyester brushes exhibited different hydrolysis kinetics and mechanisms. PHB brushes were susceptible to degradation at all pH ranges, whereas PLA and PCL were relatively stable at neutral pH but degraded under basic conditions. By AFM morphology imaging of the brushes during degradation, we elucidate the differences in the erosion mechanism occurring for each type of brush. Our results indicate that evaluating the type of degradation undergone by brushes is not trivial. Although they have a reduced thickness in comparison to conventional polymer films, bulk or surface erosion may occur depending on the polymer brush type and the pH of the surrounding media.

Furthermore, we prove the reusability of the macroinitiator-coated surfaces by the repeated growth of polyester brushes from previously degraded samples. This way, we enable the modification of surfaces with well-defined and degradable brush coatings, which can be repeatedly grown on the same surfaces, leading toward the implementation of circular polymer brush coatings.

## Experimental Section

### Materials

Silicon wafers (100.0 ± 0.5 mm diameter and 525 ± 25 μm thickness, boron-doped with (100) orientation, 5–10 Ω cm, Okmetic) were cut in 1 × 1 cm sample sizes and were used as a substrate for polymer brush growth. 1,8-Diazabycyclo[5.4.0]undec-7-ene (DBU, 98%, Merck) was distilled from calcium hydride and stored over molecular sieves (3 and 4 Å) under a nitrogen atmosphere at −20 °C. 1,5,7-Triazabicyclo[4.4.0]dec-5-ene (TBD, 98%, Merck) was used as received and stored under a nitrogen atmosphere at −20 °C. Racemic lactide (*r*-lactide, LA) was recrystallized three times from toluene and stored at −20 °C. ε-Caprolactone (ε-CL, 98%, Merck) and β-butyrolactone (β-BL, 98%, Merck) were distilled from calcium hydride and stored at −20 °C. Copper(I) bromide (CuBr, Merck, 98%) was purified by three washing cycles in acetic acid and ethanol. After that, it was dried overnight in a vacuum oven at room temperature.

(3-Aminopropyl)triethoxysilane (APTES, 99%), poly(glycidyl methacrylate) (PGMA, Mn = 10 kDa), hydrogen peroxide (H_2_O_2_, 30%), tris(hydroxymethyl) aminomethane (TRIS, ACS reagent, ≥98%), toluene (anhydrous, 99.8%), methyl ethyl ketone (MEK, ACS reagent, ≥99.5%), *N*,*N*-dimethylformamide (DMF, ≥99.8%), tetrahydrofuran (THF, anhydrous, ≥99.9%, inhibitor-free), α-bromoisobutyryl bromide (BiBB, 98%), triethylamine (TEA, ≥99%), 2-methacryloyloxyethyl phosphorylcholine (MPC, 97%), 2,2′-bipyridyl (≥99%), copper(II) bromide (CuBr_2_, ≥99%), chloroform-*d* (CDCl_3_, 99.8 atom % D), dichloromethane (DCM, ACS reagent, ≥99.5%), sodium carbonate (NaHCO_3_, powder, ≥99.5%, ACS reagent), and sodium hydroxide (NaOH, ACS reagent, ≥97.0%, pellets) were purchased from Merck and used as received. Sulfuric acid (95–97%), methanol (AR), and acetone (AR) were purchased from Biosolve and used as received. Milli-Q water was purified from a Milli-Q Advantage A10 purification system (Merck Millipore, Billerica, Ma, USA). Artificial seawater was prepared by the addition of nano reef salt (34.0 g, 35% salinity, with Na ∼ 11,000 mg/L, Mg ∼ 1200 mg/L, Ca ∼ 420 mg/L, K ∼ 350 mg/L, Cl ∼ 19,700 mg/L, SO4 ∼ 2200 mg/L, HCO3-/CO3 ∼ 180 mg/L, and other traces, Aqua Medic GmbH, Germany) to a total volume of 1 L deionized water. A pH electrode was used for the determination of the solution’s pH to 8.5, adjusted by the addition of a sodium hydroxide solution (NaOH 2 M, 0.7 mL). Phosphate buffered saline solutions (0.1 M phosphate buffer, 0.027 M potassium chloride, and 1.37 M sodium chloride, pH 7.4, tablets, Merck) of pH 7.5 and pH 8 were prepared, adjusting the pH by the addition of 1 M hydrochloric acid (HCl, ACS reagent, 37%, Merck) or sodium hydroxide (2 M, Merck) solutions. Acetate buffer (pH 4, 0.1 M) was prepared by mixing sodium acetate (ACS reagent, ≥99%, Merck) and acetic acid (ACS reagent, ≥99.8%, Merck) solutions.

### Methods

#### Macroinitiator Preparation

**Figure 1:**
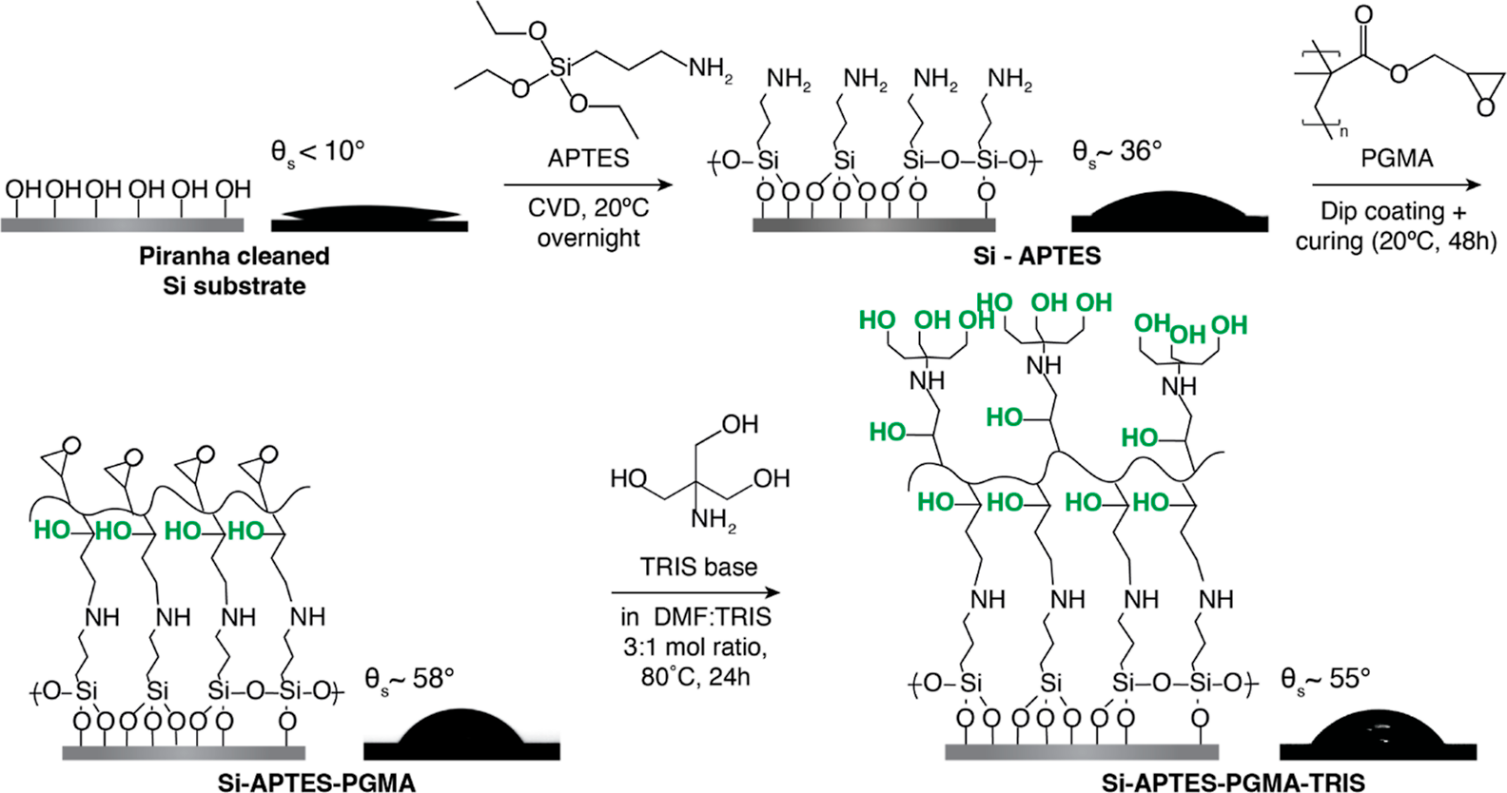
Macroinitiator preparation for polymer brush growth. Droplets show static water contact angles (θ_s_) after each deposition step.

A schematic representation of this process is shown in Figure 1. Briefly, silicon substrates were thoroughly cleaned in a piranha solution (H_2_SO_4_/H_2_O_2_ = 3:1 v/v %) and subsequently rinsed with water and ethanol and dried with nitrogen. Then, APTES was deposited onto the clean substrates via chemical vapor deposition^10,37^ to enhance the coupling with the PGMA on the substrate. After that, PGMA was covalently attached to the amino groups on the APTES layer. The substrates were immersed in a 0.1 wt % PGMA solution in MEK for 2 min, after which the wafers were cured at 20 °C for 48 h. Next, the samples were sonicated in chloroform for 1 min and subsequently rinsed with water, ethanol, and dried under nitrogen. Lastly, to increase the number of OH-groups that act as initiating sites for SI-ROP, TRIS was coupled with the remaining epoxide groups of PGMA. The samples were immersed in a 2 wt % TRIS solution in DMF under constant stirring (300 rpm) at 80 °C for 24 h, after which they were rinsed with methanol, water, and ethanol, and dried under nitrogen.

#### Organocatalytic Surface-Initiated Ring-Opening Polymerization

**Figure 2:**
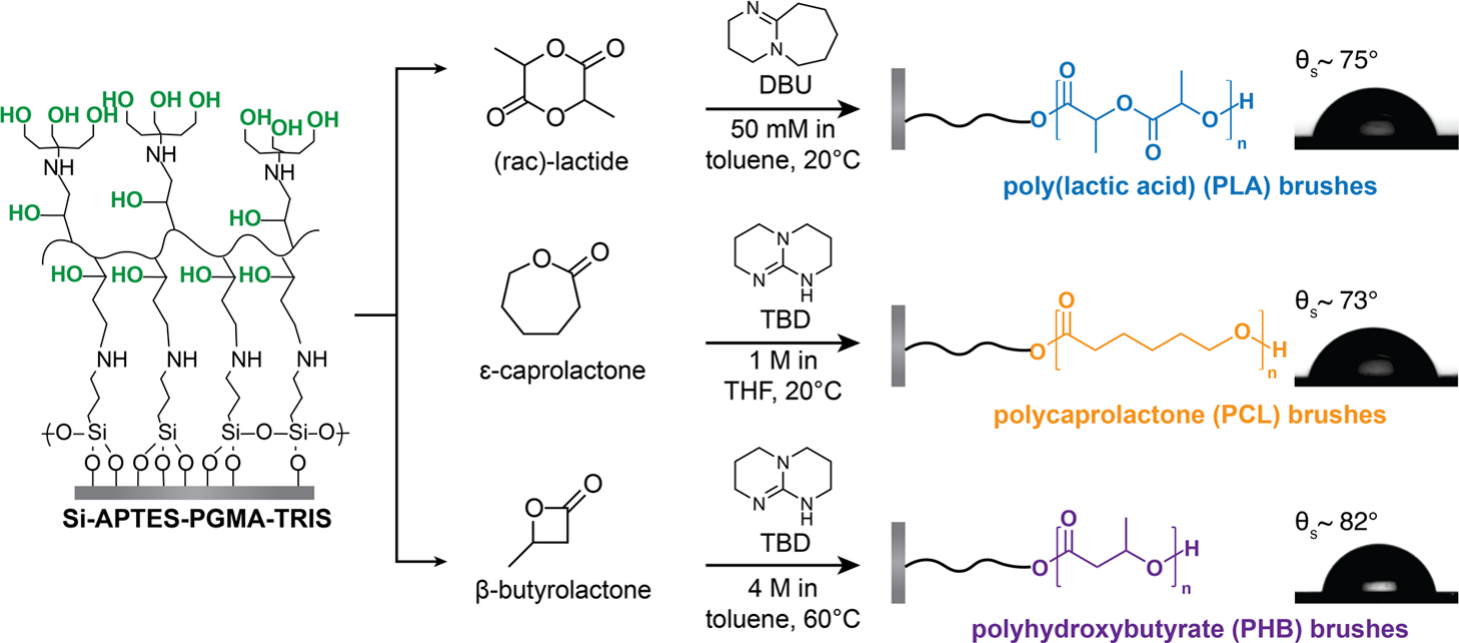
Organocatalytic SI-ROP of lactones to form polyester polymer brushes. Droplets show the static water contact angles (θ_s_) of each polyester brush.

The macroinitiator-functionalized silicon wafers were dried at reduced pressure overnight to remove ambient moisture or any possible solvent traces on the surface. All polymerizations were conducted in a glovebox (MBraun, Unilab, O_2_ and H_2_O < 0.1 ppm). A schematic representation of the polymer brush growth process is shown in Figure 2. The synthetic approach for polyester brush growth was adapted from bulk polyester polymerizations, adjusting the type and ratios of catalyst and omitting the addition of initiator in solution.^33,42,43^

#### PLA Brushes

Functionalized wafers were placed in a 20 mL glass vial with a septum cap, where *r*-lactide (0.04 g, 0.35 mmol) was mixed with toluene (5 mL) under constant stirring. Once dissolved, DBU (3 μL, 0.03 mmol) was quickly added to the vial under constant stirring. The reaction was carried out for 2 h at room temperature, after which it was stopped with a few drops of a 20 mg/mL formic acid solution in DCM. The wafers were then washed with toluene, water, ethanol, and dried under a nitrogen stream.

#### PCL Brushes

The functionalized wafers were placed in a 20 mL glass vial with a septum cap, where ε-caprolactone (0.44 mL, 4 mmol) was mixed with THF (2 mL) under constant stirring. In a second vial, TBD (3.3 mg, 0.02 mmol) was dissolved in THF (2 mL) under vigorous stirring. Once dissolved, the solution was transferred to the first vial. The reaction was carried out for 18 h at room temperature, after which it was stopped with a few drops of formic acid solution. The wafers were then washed with THF, water, ethanol, and dried under a nitrogen stream.

#### PHB Brushes

Functionalized wafers were placed in a 20 mL glass vial with a septum cap, where β-butyrolactone (1.6 mL, 20 mmol) was mixed with toluene (2 mL) under constant stirring. In a second vial, TBD (28 mg, 0.2 mmol) was dissolved in toluene (3 mL) under vigorous stirring. Once dissolved, the TBD solution was transferred to the first vial. The reaction was carried out for 20 h at 60 °C, after which it was stopped with a few drops of formic acid solution. The wafers were then washed with THF, water, ethanol, and dried under a nitrogen stream.

#### Characterization of Polyester Brushes

Hydrophilicity changes were evaluated via static contact angle measurements using an optical contact angle goniometer (OCA15, Dataphysics, Germany). Three water droplets were captured per sample, and the drop contour was fitted with the Young–Laplace equation. FTIR measurements were conducted on a Bruker Alpha spectrometer to confirm successful brush growth by the appearance of characteristic absorbance bands. A bare silicon substrate was first used to generate the background spectrum. AFM imaging of the polymer brush morphology was performed on a Multimode 8 AFM, using a NanoScope V controller and JV vertical scanner (Bruker, USA). PeakForce Quantitative Nanomechanical Mapping (PF-QNM) was the operational mode. Aluminum-coated cantilevers of a nominal spring constant of 42 N·m^–1^ and a nominal tip apex radius of 8 nm (NCH, NanoWorld, Switzerland) were used. During imaging, scanning parameters were automated using ScanAsyst Control (NanoScope, v.9.7), to ensure well-controlled imaging with a minimal applied load. Resulting images were analyzed with NanoScope Analysis software (version 2.0).

SE was performed on an M-2000X spectroscopic ellipsometer (J.A. Woollam, USA) operating at a wavelength range from 245 to 1000 nm, with 5 s sampling time at three angles of incidence (65, 70, and 75°). Data fitting and analysis was performed using CompleteEASE integrated software, where an optical model comprising of a Si substrate, a 1 nm native SiO_*x*_ layer, and a Cauchy layer were used to determine brush thicknesses. Gel permeation chromatography (GPC) was conducted on an Agilent 1260 infinity system, equipped with a G1322A degasser, G1310B iso pump, G1329B 1290 infinity autosampler, G7116B MCT oven, G1314F VWD detector, G1362A RID detector, a Wyatt Viscostar-II viscometer, and Minidawn TREOS light scattering detector. Columns: PSS SVD-10000A 3um + SVD-50A 3um +50A guard column with chloroform as the eluent. Molecular weights were calculated from these measurements by using PSS calibration standards.

#### Grafting Density Evaluation

Polymer brush conformations on surfaces depend greatly on the relation between the molecular weight of the polymer chains and their grafting density (σ).^13^ Theoretically, this follows eq 1
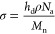
1where *h*_d_ is the dry thickness of the brush layer, ρ is the bulk density of the studied polymers, *N*_a_ being Avogadro’s number, and *M*_n_ is the number-average molecular weight. Calculating σ of grafted brushes grown via grafting from methods remains challenging due to the unknown *M*_n_ of the layers prior to the synthesis of the layers. However, this is possible by using cleaving agents, which can hydrolyze the bonds between the solid surface and the polymer chains with negligible effects on the polymer main chain. Considering the labile ester bonds present in our brushes, a mild TBAF cleaving agent solution was used for the cleaving of the layers to analyze their molecular weight without compromising the main chain of the studied polyester brushes.^44,45^

Silicon wafers coated with polyester brushes of 40–50 nm thickness were placed in a flat bottomed flask together with 25 mL of a 0.04 M TBAF solution in THF.^46^ An increased wafer size (4 samples of 5 × 2 cm^2^) was used in these experiments to ensure a sufficient polymer concentration for analysis resulting from cleaving the chains present on the surface with TBAF. The samples were incubated for 24 h at 55 °C. After that, the wafers were removed from the solution, rinsed with THF, water, and ethanol, and dried under nitrogen. After the cleaving treatment, a layer thickness of 2 nm was measured with ellipsometry, indicating a complete brush detachment together with the PGMA macroinitiator. In parallel, the THF cleaving solution was evaporated, and the TBAF was separated by redissolving in water and precipitating the polyester polymers. The isolated cleaved polymer brushes were then dried and redissolved in chloroform for GPC analysis.

#### Degradation Kinetics

Polymer brush-coated silicon substrates were immersed in 10 mL buffer solutions of different pH (4, 7.5, and 8, respectively) and artificial seawater (pH 8). At various time intervals, the wafers were removed from the flasks, minimally rinsed with DCM, water, and ethanol, and dried under a nitrogen stream. After that, thickness measurements were performed by ellipsometry.

## Results and Discussion

### Macroinitiator Preparation and Stability

**Figure 3:**
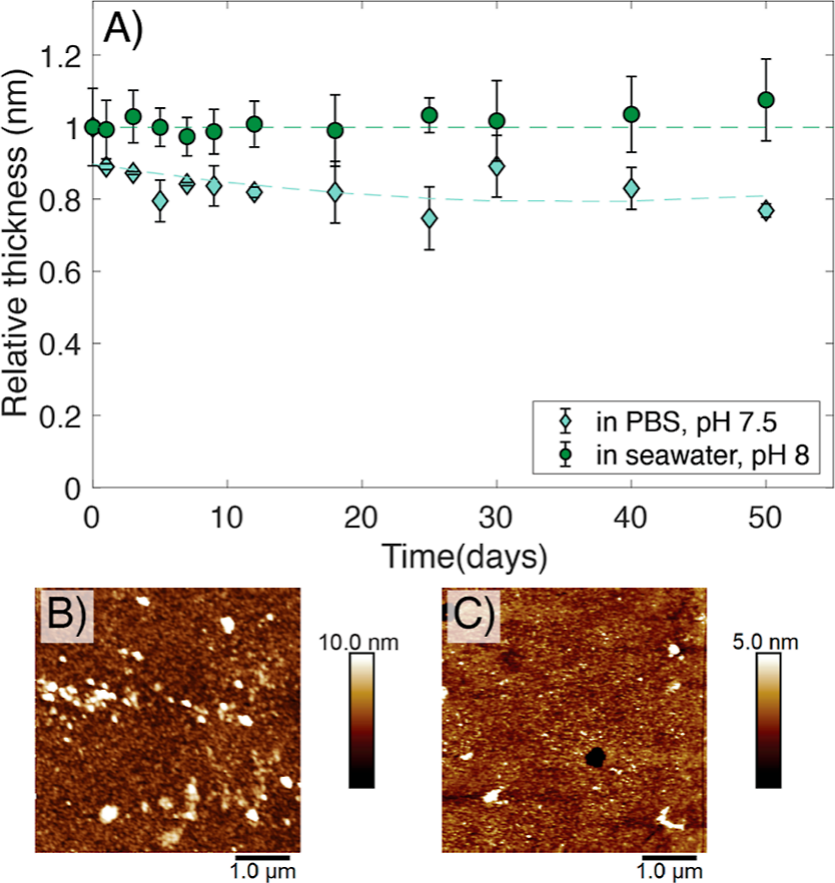
(A) Relative thickness profile over incubation time of the macroinitiator in PBS buffer solution (pH 7.5, diamonds) and seawater solution (pH 8, circles). The initial thickness of the macroinitiator was 8 ± 2 nm. AFM images depict the morphology of the macroinitiator layer after 50 days incubation in (B) PBS pH 7.5 and (C) seawater.

For the synthesis of polyester brushes, we followed a two-step approach, including the preparation of a stable macroinitiator layer (Figure 1), which was used for the SI-ROP of lactones (Figure 2). In short, silicon wafers were coated with a hydroxyl-terminated macroinitiator, which poses as a surface anchor and initiating layer for polyester brush growth. The macroinitiator consists of an APTES layer, which is bound by chemical vapor deposition to the silicon substrate. APTES was utilized to improve the binding homogeneity of the next PGMA layer to the surface and to eliminate the common curing step for PGMA binding. Then, we increase the number of initiating OH- groups by the reaction of PGMA with TRIS base. Although TRIS has been similarly reacted with PGMA polymers,^47^ we show the first report of its synthesis and use as a macroinitiator for brush growth. Static contact angle measurements were used as a qualitative indication of a successful macroinitiator deposition. The APTES-modified silicon substrate exhibited a contact angle of 36°; after the reaction with PGMA, an increased hydrophobicity (58°) was measured, which remained relatively unchanged (55°) after the reaction with TRIS to the final macroinitiator layer. SE measurements proved no changes in the thickness of the macroinitiator-layer over a period of at least 50 days after immersion in seawater; in PBS ca. 15% loss of thickness was determined after 50 days (Figure 3A).

The morphology of the macroinitiator remained unchanged for 50 days when immersed in PBS solutions, as shown by AFM images (Figure 3B). In seawater, a slight increase in roughness was observed after 25 days. This was also shown by an increased deviation in surface thickness measured by ellipsometry and the formation of small (0.4 μm diameter, 3.5 nm depth) perforations that appear throughout the surface of the macroinitiator shown by AFM images (Figure 3C). These results show that no degrafting of the macroinitiator layer takes place, which is an important prerequisite for studying the hydrolysis of grafted polyesters and the reusability of these surfaces.

To further investigate the stability of the macroinitiator layer, we grafted a well-known, water-soluble, and zwitterionic polymer brush, poly(2-methacryloyloxyethyl phosphorylcholine) (PMPC), from the modified silicon surfaces via surface-initiated atom transfer radical polymerization (SI-ATRP) (see Supporting Information for synthetic details). By grafting PMPC brushes from our macroinitiator, we obtained brushes 7 times thicker (150 nm) than the ones grown from only PGMA macroinitiators (∼20 nm).^41^ The stability of PMPC brushes in PBS solutions of pH 7.5 was evaluated, showing a moderate thickness loss of 19% after 50 days of incubation (Figure S1). These results show that our macroinitiators are also suitable for the growth of hydrophilic ATRP brushes, enabling the growth of thick hydrophilic brushes with enhanced stability.

### Synthesis and Characterization of Polyester Brushes

Polyester brushes were synthesized via organocatalytic SI-ROP from macroinitiator-coated silicon wafers. We used the commercial superbases DBU (for lactic acid) or TBD (for caprolactone and butyrolactone) as the respective organocatalysts, providing metal-free polymer brush samples with high reproducibility. By utilizing organocatalysis and moderate polymerization temperatures, we synthesized PLA brushes up to 4 times thicker than previous reports.^16,33^ Regarding PCL, although others have shown successful brush growth of relatively thick brushes,^17,48^ only one work studied the hydrolysis of PCL-brushes, and there, ultrathin (<10 nm) brushes were used.^32,34^ Other works studied PCL brush degradation in extreme environments, such as in organic solvents and/or highly alkaline solutions (pH 14).^17^ Successful and reproducible organocatalytic polymerizations of PHB have shown to be challenging in bulk, due to the formation of intermediate species which hinder the promotion of the ring opening of butyrolactone.^43^ In this work, we report the first PHB grafted polymer brushes.

**Figure 4:**
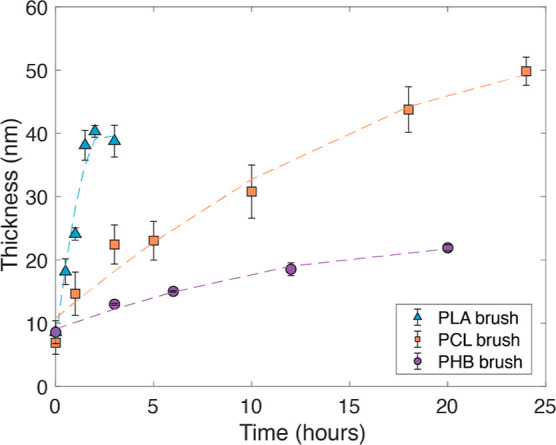
Kinetics of the SI-ROP of LA, ε-CL β-BL, 98%, measured by ellipsometry, showing the increase of brush thickness over polymerization times. Error bars denote the standard deviation with a 95% confidence interval.

We analyzed the kinetics of the SI-ROP for all systems by ellipsometry, showing a steady increase of brush thickness over time (Figure 4) that reflects the controlled nature of the ROP polymerizations. After a short polymerization time of 2 h, we obtained PLA brushes of 40 ± 1 nm thickness. Similar thicknesses were obtained for PCL brushes after longer polymerization times (24 h led to 49 ± 2 nm brushes). PHB brushes grew up to 22 ± 0.5 nm after 20 h of polymerization.

Under the chosen conditions, the relative kinetics of SI-ROP followed similar trends as for solution polymerization, with PLA being very fast when using DBU catalysis, while PCL and PHB revealed slower growth kinetics, even when the stronger organobase TBD was applied. When using DBU as an organocatalyst, both caprolactone and butyrolactone did not produce polymer brushes.

**Figure 5:**
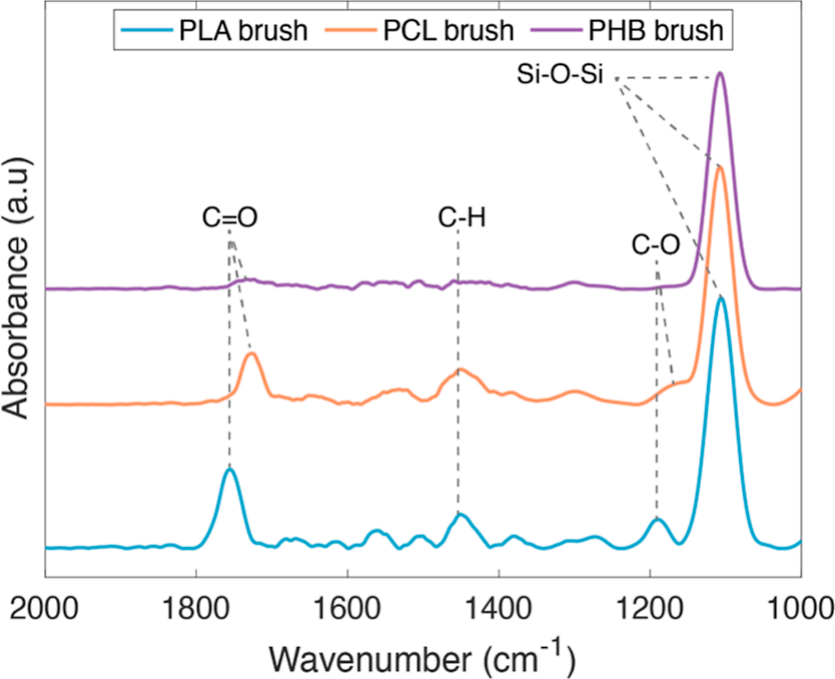
FTIR reflection spectra of silicon substrates coated with PLA, PCL, and PHB polyester brushes.

FTIR measurements were used to confirm the formation of the brushes (Figure 5). Together with the thickness profiles and morphology imaging, the presence of the peaks of the C–O stretch at 1190 cm^–1^, carbonyl stretch at 1750 cm^–1^, and C–H bending at 1450 cm^–1^ indicates strongly that these polyesters have been grafted from the surface (Si–O–Si stretching of the underlying surface is located at 1100 cm^–1^). The reduced thickness of the PHB brushes in comparison to the PLA and PCL brushes is reflected in the lower intensity of their characteristic absorbance peaks.

**Figure 6:**
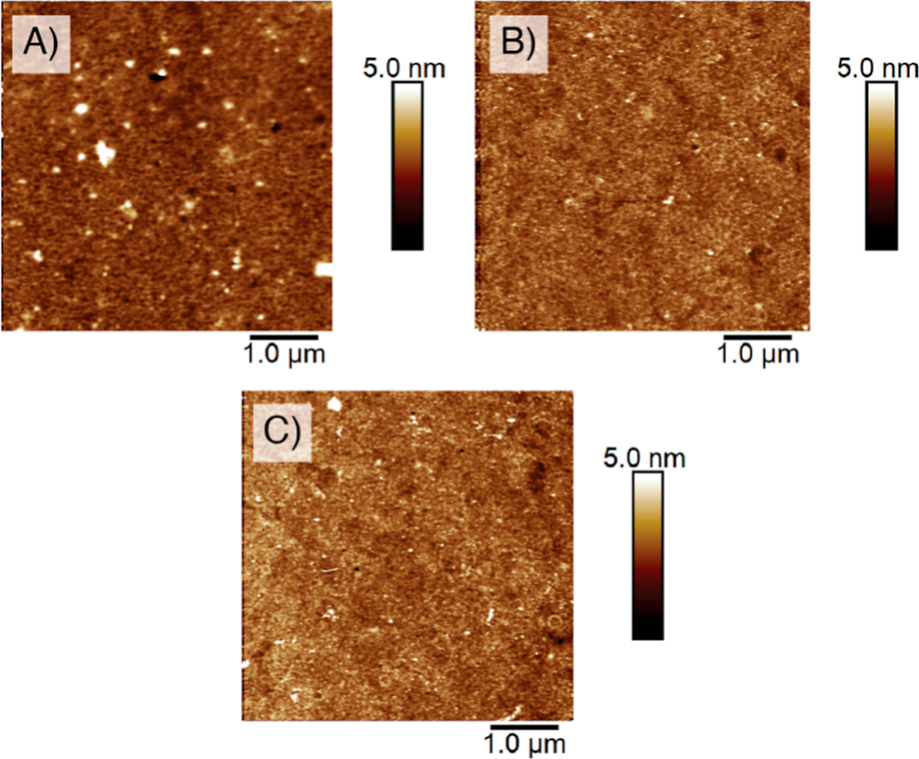
AFM initial morphology images of (A) PLA, (B) PCL, and (C) PHB brushes.

We confirm the presence of dense and homogeneous brush films by AFM imaging (Figure 6A–C). Low-density brushes would show a nonhomogeneous surface coverage and high roughness, as it has been previously reported.^49^ From these AFM images, we can also assume that the polymer chains are present in an amorphous state, due to the lack of periodical structuring throughout the layer.^50^ Our brushes exhibited a regular and homogeneous morphology, with a reduced root-mean-square roughness (*R*_q_) of 1.20, 0.85, and 0.82 nm for PLA, PCL, and PHB, respectively.

To determine the molar mass and grafting density, PLA and PCL brushes of 40–50 nm in thickness were cleaved from their respective silicon surfaces using TBAF^44,46^ and the resulting polymer was analyzed by GPC. Cleaved PLA brushes showed peaks with *M*_n_ = 239 kDa and moderate molecular weight distribution (*M*_w_/*M*_n_ = 1.29, Figure S2A). Using eq 1, the PLA brush grafting density was estimated as σ = 0.12 chains/nm^2^. Cleaved PCL brushes showed an Mn of 229 kDa and an *M*_w_/*M*_n_ of 1.32 (Figure S2B), leading to an estimated grafting density of σ = 0.11 chains/nm^2^. These grafting density estimates are in agreement with previous reports of moderately dense hydrophobic brushes grown via grafting from ref (10).

Due to the reduced thickness of the PHB brush coatings, it was not possible to obtain enough sample volumes for GPC analysis. For these brushes, the free PHB polymer grown in solution during brush polymerization was analyzed by GPC. These showed an *M*_n_ = 5 kDa, with an *M*_w_/*M*_n_ = 1.4, (Figure S2C), leading to an estimated grafting density of σ = 2.2 chains/nm^2^. We would expect a higher molecular weight and thus lower grafting density for the grafted brushes in comparison to the residual polymer in solution, as it has also been similarly shown in previous works.^16^

### Degradation Studies

**Figure 7:**
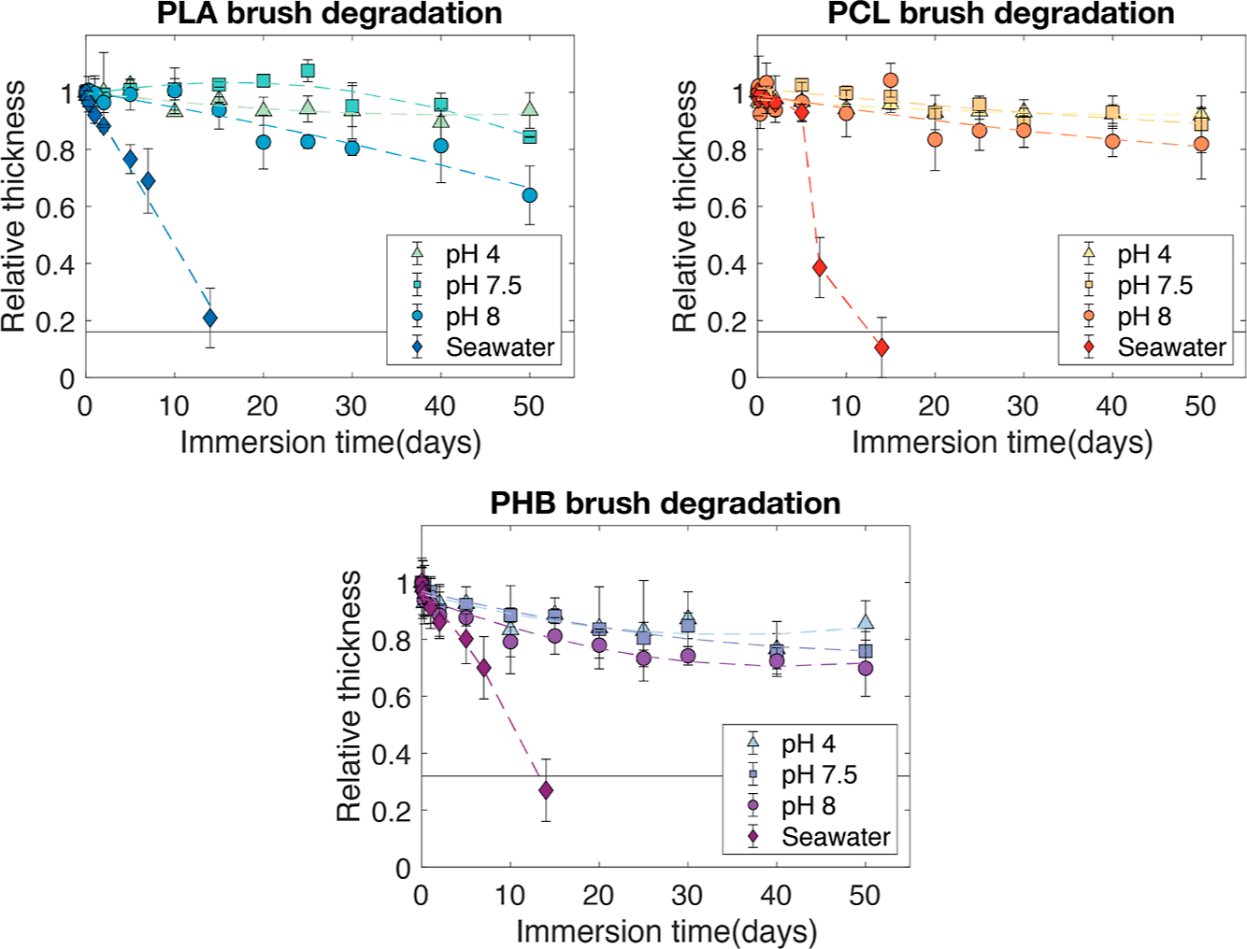
Degradation profiles of PLA, PCL, and PHB brushes measured by ellipsometry. The normalized brush thickness was evaluated after repeated immersion in buffered solutions and seawater. The horizontal line represents the relative thickness occupied by the macroinitiator. Error bars denote the standard deviation with a 95% confidence interval.

The kinetics and extent of degradation of each polyester brush after repeated incubation in buffered solution of pH 4, 7.5, 8, and seawater of pH 8.5 were evaluated via SE and fitted with a Cauchy model (Figure S3 showing 15 days incubation and Figure 7 showing 50 days incubation). PLA brushes remained stable for the first 15 days incubated in solutions of up to pH 8. A 2.5% thickness loss was observed in pH 4, with an average degradation rate of 0.08 nm/day. That was followed by a 4.8% loss in pH 7.5 at 0.1 nm/day and 6.2% in pH 8.5 at 0.2 nm/day. PCL brushes showed a slightly higher stability during the first 15 days of incubation in the studied media. A 0.9, 4, and 7% thickness loss was observed in solutions of pH 4, pH 7.5, and pH 8, at an average degradation rate of 0.03, 0.08, and 0.25 nm/day, respectively. PHB brushes showed earlier degradation signs in comparison to the other two studied polyesters, having a 12, 15, and 18% thickness loss in pH 4, 7.5, and 8, respectively. For these brushes, average degradation rates of 0.17, 0.17, and 0.23 nm/day were observed (Figure S3).

Interestingly, the degradation was rapid in seawater, where all polyester brush coatings were totally removed after 14 days of incubation. We attribute this change in degradation profiles to the difference in ionic strength of the buffered solutions (0.1 M) in comparison to seawater. PLA and PHB brushes showed a gradual decay of their thicknesses of 2.7 and 1 nm/day, respectively. PCL brushes showed two different degradation stages in seawater, having a slow and linear degradation for the first 5 days, followed by an abrupt decay and total loss of the coating. In the first 5 incubation days, the coating lost 7% of its initial thickness at a slow degradation rate of 0.66 nm/day. In the next 2 days, the coating lost 54% of its thickness, followed by its total removal under 15 days of incubation. Degradation in seawater of PLA, PCL, and PHB has been studied for bulk samples in multiple works.^51^ There, for the same degradation times as the ones of this study and longer, no degradation occurred. Only PHB bulk samples showed a weight loss of ∼8% after one year incubation in seawater.^23^ We attribute the enhanced degradation of our brushes to the reduced layer thickness when compared with bulk samples and the higher accessibility of the end-groups in the polymer brush chains, making them susceptible to hydrolysis.

Degradation was further studied for all brushes in buffered solutions up to pH 8 (Figure 7). After the first 15 days of incubation, degradation processes became faster for PLA brushes, having a stronger thickness decay at an increasing pH of the solution. After 50 days, PLA brushes did not show further signs of degradation at pH 4 (2.5% loss), followed by a 16% loss at pH 7.5. The strongest degradation signs occurred at pH 8, with a 37% thickness loss after 50 days. For PCL brushes, these degradation trends were not followed. PCL brushes showed enhanced long-term stability in comparison to PLA brushes, with a maximum loss of 19% relative thickness at pH 8 after 50 days of incubation. Lastly, PHB brushes were the most susceptible to degradation, though they were not fully removed after 50 days of incubation in solutions of up to pH 8, where there was a maximum thickness loss of 30%. An overview on degradation rates for each polyester brush at varying pH in solution is shown in Table S1. By means of contact angle, we also observed degradation signs by decays in contact angles for all brushes at increased pH, which are related to the enhanced thickness losses (summarized in Table S2).

To understand the degradation mechanism of our polyester brush coatings, we evaluated changes in the morphology of the brush layer over incubation time. By combining the degradation profiles of each brush in all the studied media and the changes in morphology by AFM, we elucidated the erosion mechanism undergone by the brushes. After incubation in acidic media (pH 4), PLA and PCL brushes showed a slight increase in roughness (Figure S4). Together with minimal hydrolysis and thickness loss for both brushes after 50 days of incubation, we attribute this as a bulk erosion mechanism. PHB brushes showed earlier degradation signs in acidic media together with morphology changes due to faster hydrolysis, indicating a surface erosion mechanism.

**Figure 8:**
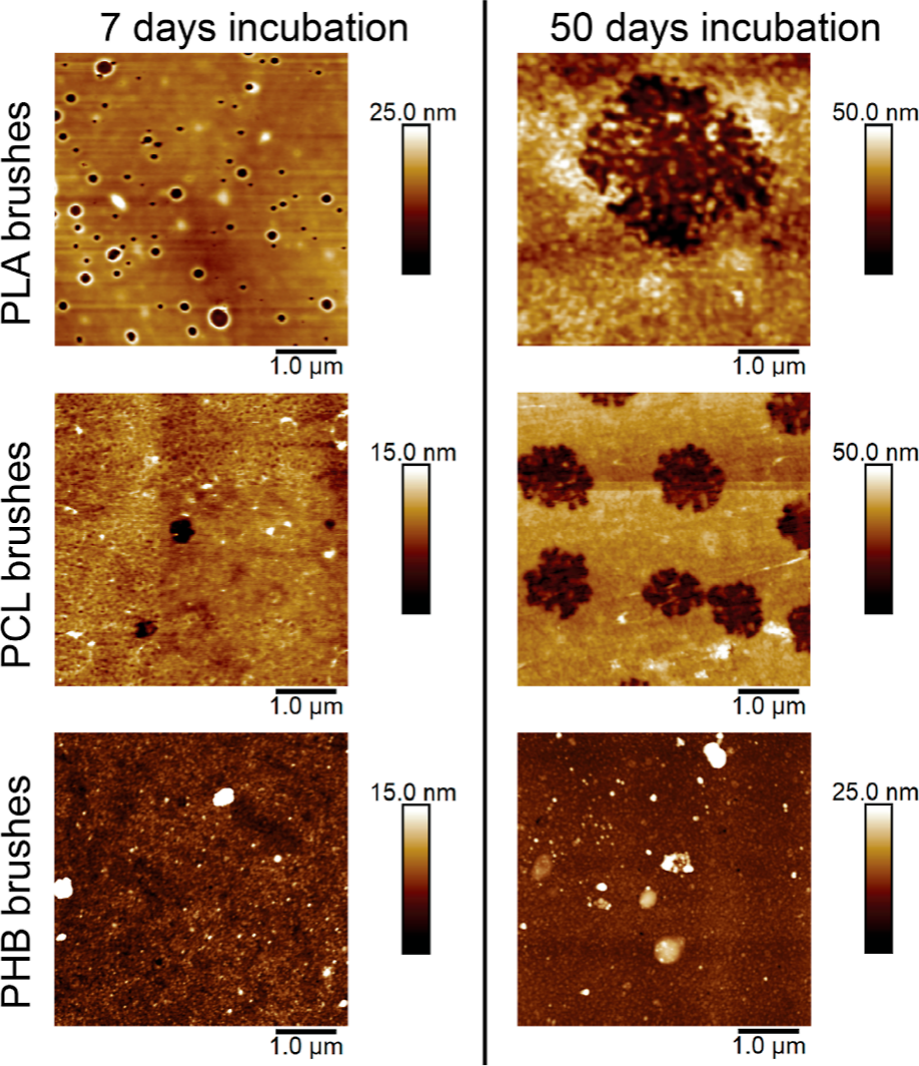
AFM morphology images of PLA (top), PCL (middle), and PHB (bottom) polymer brushes after 7 (left) and 50 (right) days in a PBS solution of pH 7.5.

When incubated in buffered solutions of higher pH (7.5), we observed a change in morphology, which we attribute to a shift in erosion mechanism from bulk to surface erosion for PLA and PCL brushes. This is shown by the formation and growth of voids along the coating. These voids do not only remain within the AFM sample size (5 μm), but throughout the entire coated sample (1 cm^2^). This occurred earlier for PLA brushes than for PCL brushes, appearing at a higher concentration after 5 days of incubation for PLA (Figure 8 top and middle row, respectively). After 50 days of incubation, there was an increase in void size for both brushes, with an average depth of 7 nm for PLA brushes and 4 nm for PCL brushes. These void depths correspond to the thickness losses shown in the ellipsometric degradation profiles in Figure 7. In pH 7.5, PHB brushes showed great changes in surface morphology, having a surface roughness increase at increasing incubation times (Initial *R*_q_ = 0.82 nm, *R*_q,5days_ = 2.8 nm, and *R*_q,50days_ = 3.9 nm). The increase in surface roughness, together with a fast hydrolysis rate, indicates that PHB brushes also undergo surface erosion at pH 7.5.

When incubated at pH 8, great changes in morphology indicated by a roughness increase and layer inhomogeneities were observed (Figure S4, third column). For PLA and PCL brushes, the formation of voids throughout the surface was observed but was not predominant. Instead, PLA brushes showed an increase in surface roughness of ∼20 nm, with PCL and PHB having a roughness increase of ∼8 nm. These findings indicate that at pH 8, due to a faster hydrolysis rate, as also indicated by the degradation profiles, hydrolysis became faster than the water diffusion throughout the brush. This is an indicative of a surface erosion mechanism. These effects were enhanced when incubated in seawater, where an abrupt increase of surface roughness for all samples was observed, after which the layer could be removed during the rinsing step after 14 days of incubation (Figure S4, right column).

**Figure 9:**
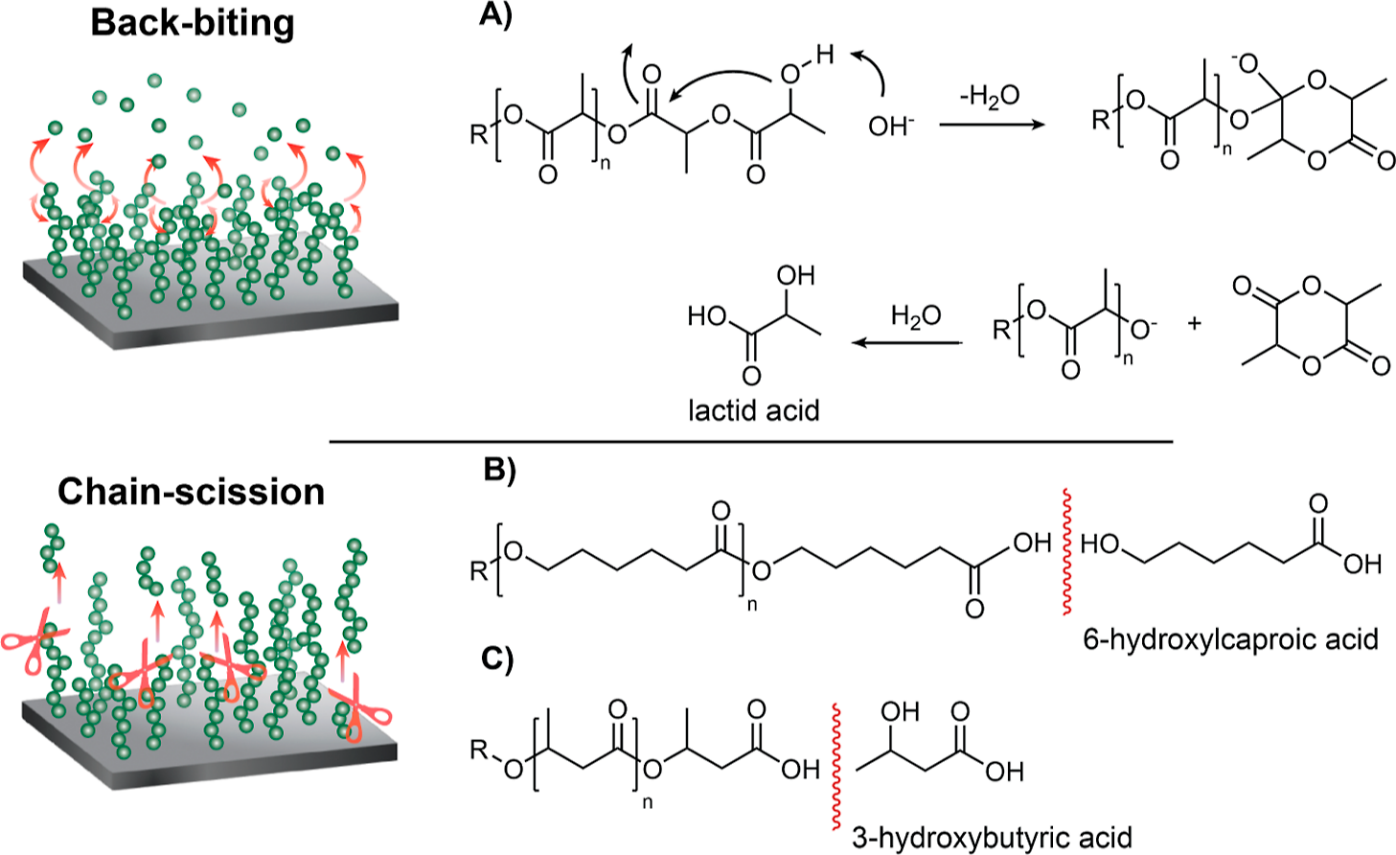
Proposed degradation mechanisms of polyester brushes in solution, showing schematic representations of (A) PLA, (B) PCL, and (C) PHB hydrolysis.

Considering the degradation profiles and the erosion mechanisms observed via morphology changes for each brush at the studied pH ranges, we propose distinct degradation mechanisms, shown in Figure 9. The overall slow though gradual degradation shown by PLA brushes suggests that backbiting mechanisms were predominant. For these brushes, degradation is base-catalyzed but does not occur in acidic media, similarly to how the same polymers degrade in bulk. PLA oligomers also degrade in acidic media, which was not shown in our study.^16^ We assume that PCL brushes undergo a chain scission mechanism due to the much slower degradation in comparison to PLA up to pH 8. In the case of PHB brushes, degradation occurs at all of the studied pH ranges to a certain extent, which is common of chain scission mechanisms. Our results are in agreement with previous works that suggest similar degradation trends for both PLA and PCL brushes. However, in comparison to previously reported polyester brushes, ours show improved durability due to their higher thickness and strong binding to the surface by the PGMA-based macroinitiator.^16,34^

### Surface Reusability

We prove the reusability of the macroinitiator-coated surfaces by the repeated growth of polyester brushes from previously degraded samples. Silicon wafers previously coated with PHB brushes were used for regrowth evaluation after they were incubated for 50 days in seawater. We selected these samples due to their enhanced degradation effects in comparison to those of PLA and PCL brushes. The surfaces were sonicated in chloroform for 10 min, after which they were dried under reduced pressure for 24 h. PLA brushes were then grown from the already used surfaces following the synthetic methodology previously described together with a silicon wafer coated with fresh macroinitiator.

**Figure 10:**
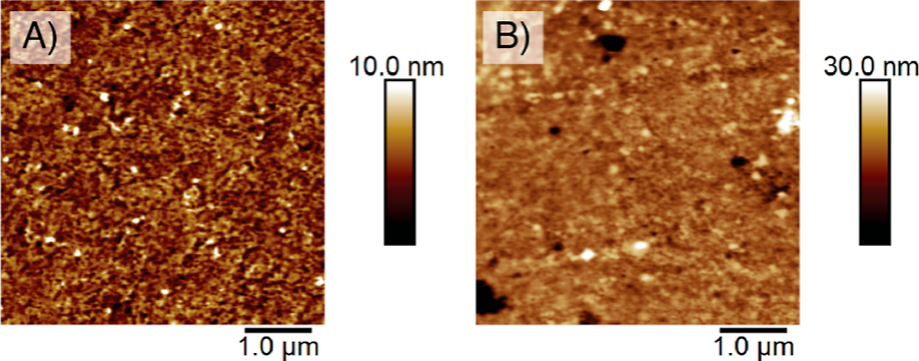
AFM morphology images of (A) PLA brushes grown from fresh macroinitiators and (B) PLA brushes grown from reused macroinitiators.

This way, PLA brushes of 37 nm were grown from reused macroinitiators as measured by ellipsometry. In comparison, the same brushes grown from fresh macroinitiators (blank) were 45 nm. A slight increase in surface roughness was observed for the PLA brushes grown from reused surfaces, with root-mean-square roughness of *R*_q_ = 3.6 nm, compared to a *R*_q_ = 1.3 nm for the blank PLA brushes (Figure 10). Our results show the excellent performance of the macroinitiator, which maintains its initiating character after long exposures to basic solutions. This way, we enable the modification of surfaces with well-defined and degradable brush coatings which can be repeatedly grown on the same surfaces, leading toward the implementation of circular polymer brush coatings.

## Conclusions

We have shown a systematic study of the synthesis and degradation of polyester polymer brushes. The use of organocatalysts and moderate temperatures were optimal for the surface-initiated ring opening polymerization to grow PLA, PCL, and PHB brushes. By using a PGMA-based macroinitiator, we ensure that polymer chain degrafting is minimized. This way, we quantified the inherent polymer degradation by spectroscopic thickness evaluation over time. Our degradation studies indicate a strong dependency on the pH, with more pronounced thickness losses at increased basicity and no degradation occurring at pH 4.

Distinct erosion mechanisms were observed per brush when the brush was incubated in acidic or basic media by AFM imaging. Here, a shift from bulk to surface erosion at pH 7.5 was observed for PLA and PCL brushes. Together with the degradation kinetic profiles, we attribute different degradation mechanisms for each brush, being backbiting for PLA and chain scission for PCL and PHB.

Interestingly, all brushes fully degraded under 15 days in seawater, which is drastically different from the degradation of the same polymers in bulk. This opens up possibilities on the design of truly degradable polymer coatings, posing as interesting alternatives to (meth)acrylate-based, nondegradable polymers brushes. In addition, the reusability of the surfaces was proven by repeatedly growing polyester brushes from previously degraded samples. By using these brushes, we enable surface modifications with a well-defined degradation that can be regrown on the same surface, moving toward a more circular approach on polymer brush growth.

## Supporting Information Available

Additional experimental details, methods and results, including figures on the stability of PMPC brushes in pH 7.5, GPC traces of brushes, short-term degradation profiles, AFM morphology images of polyester brushes after degradation, degradation rates, and static contact angles during degradation. (PDF)
