# Supplementary material for: Polyester Brush Coatings for Circularity: Grafting, Degradation, and Repeated Growth
Source: Macromolecules. 2023 Oct 19;56(21):8856–65. doi: 10.1021/acs.macromol.3c01601 (PMC10653273; doi:10.1021/acs.macromol.3c01601)
Supplement: Supplementary file 1 — ma3c01601_si_001.pdf [file ma3c01601_si_001.pdf]

Supporting Information: Polyester Brush Coatings for Circularity: Grafting, Degradation and Repeated Growth

Maria Brió Pérez, Mark A. Hempenius, Sissi de Beer, and Frederik R. Wurm*

Sustainable Polymer Chemistry Group, Department of Molecules & Materials, MESA+ Institute for Nanotechnology, Faculty of Science and Technology, University of Twente, P.O. Box 217, 7500 AE Enschede, The Netherlands

E-mail: frederik.wurm@utwente.nl

Phone: +31 (0)534895169

Supporting experimental details

Synthesis of PMPC brushes by SI-ATRP

To enable the growth of PMPC brushes, surfaces coated with macroinitiators were coupled with an ATRP initiator, α-bromoisobutyryl bromide (BiBB), for functionalization of the hydroxyl groups present on the surface. The macroinitiator-coated substrates were immersed in cold toluene (60 mL), followed by a drop-wise addition of triethylamine (1.12 mL) and BiBB (1 mL). The reaction was carried out for 3 hours at 20 °C under vigorous stirring (400 rpm). After that, the substrates were rinsed with ethanol, water and dried under nitrogen flow.

Next, SI-ATRP was used to grow the brushes from the modified surfaces.[1] In a round bottom flask, 2-methacryloyloxyethyl phosphorylcholine (MPC, 2 g, 6.8 mmol) and 2,2'-

bipyridyl (50 mg, 0.3 mmol) were mixed and dissolved in a MilliQ water (2 mL) and methanol (2 mL) solution under nitrogen purge and constant stirring (200 rpm) for 30 minutes. In a second round bottom flask, copper(I) bromide (CuBr, 16.2 mg, 0.1 mmol) and copper(II) bromide ($CuBr_2$, 7.5 mg, 0.03 mmol) were purged for 30 minutes together with a stirring bar. After that, the monomer solution was transferred with a purged syringe to the flask containing the copper bromide-based catalysts and vigorously stirred (400 rpm) until completely dissolved. Next, the mixture was transferred to a flask containing the substrates, which had also been previously purged with nitrogen for 30 minutes. The polymerization was allowed to proceed for 2 h at 20°C under nitrogen purging to produce 150 nm thick PMPC brushes. MilliQ water was injected into the flask to quench the reaction. Finally, the substrates were rinsed with water and ethanol to remove the physically adsorbed polymer.

Supporting Results

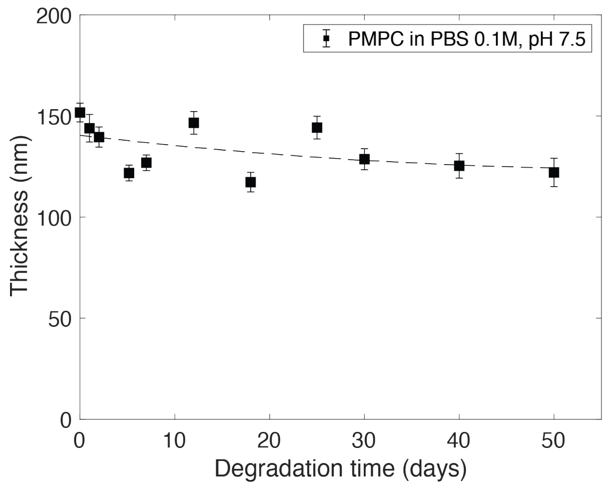

Figure S1: Stability of PMPC brushes grown from APTES-PGMA-TRIS macroinitiators in PBS 0.1 M, pH 7.5.

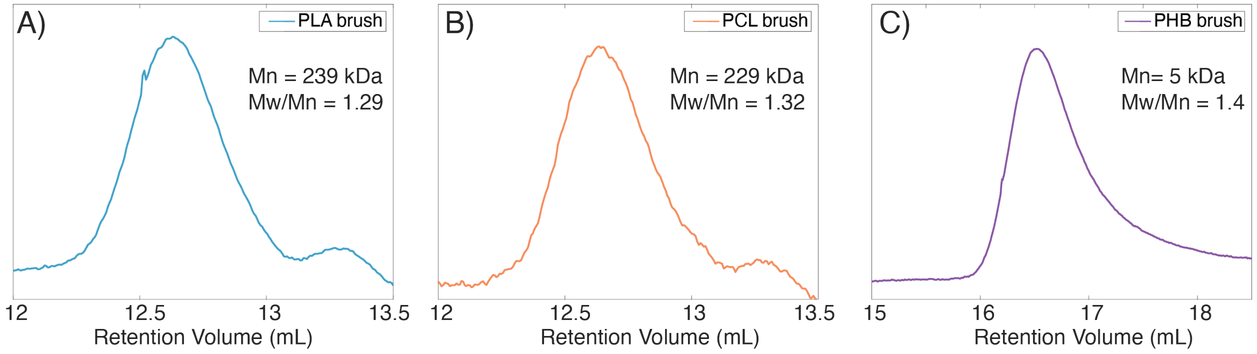

Figure S2: GPC traces of cleaved PLA (blue) and PCL (orange) brushes in chloroform. Right plot shows GPC trace of free PHB polymer grown in solution during brush polymerization.

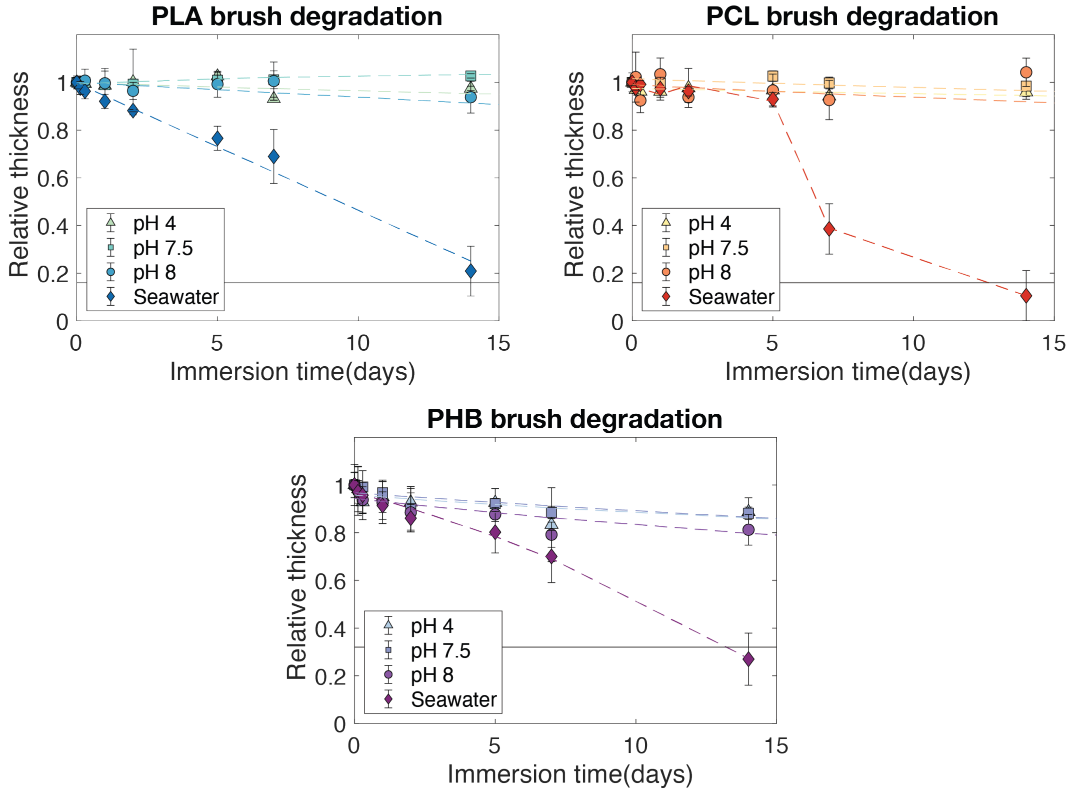

Figure S3: Short-term degradation profiles of PLA, PCL and PHB brushes measured by ellipsometry. The normalized thickness of the brushes was evaluated after repeated immersion in buffered solutions and seawater. The horizontal line represents the relative thickness occupied by the macroinitiator. Error bars denote the standard deviation with a 95 % confidence interval.

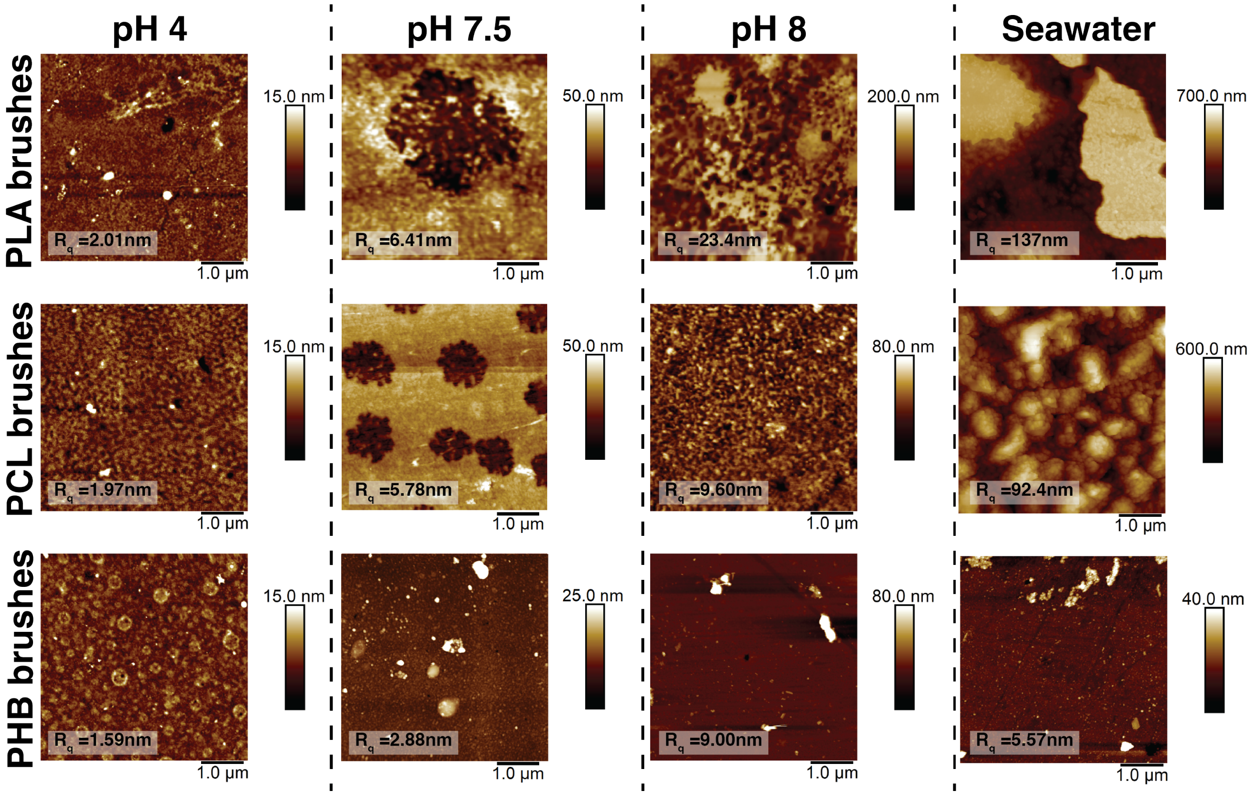

Figure S4: AFM morphology images of polyester brushes after incubation for 50 days in buffered solutions of pH 4, 7.5, 8 and for 14 days in seawater.

Table S1: Degradation rates (nm/day) and total thickness loss (%) after 50 days of incubation in the studied degradation solutions for all polyester brushes

	**PLA**		**PCL**	
Solution type	Degradation rate (nm/day)	Thickness loss (%)	Degradation rate (nm/day)	Thickness loss (%)
pH 4	0.08	6.37	0.05	4.93
pH 7.5	0.12	15.66	0.07	11.18
pH 8	0.33	36.08	0.17	18.09
Seawater	2.70	79.10	3.03	89.47

	**PHB**	
Solution type	Degradation rate (nm/day)	Thickness loss (%)
pH 4	0.06	14.44
pH 7.5	0.07	24.15
pH 8	0.11	30.14
Seawater	0.97	73.02

Table S2: Static contact angles of the macroinitiators and studied polyester brush coatings before degradation (CA_0) and after 50 days of incubation in solutions of varying pH and seawater

Surface type	CA_0 (± 5 °)	50d pH 4 (± 5 °)	50d pH 7.5 (± 5 °)	50d pH 8 (± 5 °)	50d seawater (± 5 °)
**Macroinitiator**	74	N/A	55	N/A	38
**PLA brush**	72	66	80	70	53
**PCL brush**	73	79	69	70	44
**PHB brush**	82	76	75	90	77

References

(1) Yu, Y.; Vancso, G. J.; de Beer, S. Substantially enhanced stability against degrafting of zwitterionic PMPC brushes by utilizing PGMA-linked initiators. *European Polymer Journal* **2017**, *89*, 221–229.
